# Impact of a Rapid Decline in Malaria Transmission on Antimalarial IgG Subclasses and Avidity

**DOI:** 10.3389/fimmu.2020.576663

**Published:** 2021-01-27

**Authors:** Isaac Ssewanyana, John Rek, Isabel Rodriguez, Lindsey Wu, Emmanuel Arinaitwe, Joaniter I. Nankabirwa, James G. Beeson, Harriet Mayanja-Kizza, Philip J. Rosenthal, Grant Dorsey, Moses R. Kamya, Chris Drakeley, Bryan Greenhouse, Kevin K. A. Tetteh

**Affiliations:** ^1^ Infectious Diseases Research Collaboration, Kampala, Uganda; ^2^ Department of Infection Biology, London School of Hygiene and Tropical Medicine, London, United Kingdom; ^3^ Department of Medicine, University of California San Francisco, San Francisco, CA, United States; ^4^ School of Medicine, Makerere University, Kampala, Uganda; ^5^ Burnet Institute, Melbourne, VIC, Australia; ^6^ Central Clinical School, Monash University, Melbourne, VIC, Australia; ^7^ Department of Medicine, University of Melbourne, Melbourne, VIC, Australia; ^8^ Chan Zuckerberg Biohub, San Francisco, CA, United States

**Keywords:** antibody avidity, *Plasmodium falciparum*, immunity, IgG subclass antibodies, avidity index, malaria

## Abstract

Understanding how immunity to malaria is affected by declining transmission is important to aid vaccine design and understand disease resurgence. Both IgG subclasses and avidity of antigen-specific responses are important components of an effective immune response. Using a multiplex bead array assay, we measured the total IgG, IgG subclasses, and avidity profiles of responses to 18 *P. falciparum* blood stage antigens in samples from 160 Ugandans collected at two time points during high malaria transmission and two time points following a dramatic reduction in transmission. Results demonstrated that, for the antigens tested, (i) the rate of decay of total IgG following infection declined with age and was driven consistently by the decrease in IgG3 and occasionally the decrease in IgG1; (ii) the proportion of IgG3 relative to IgG1 in the absence of infection increased with age; (iii) the increase in avidity index (the strength of association between the antibody and antigen) following infection was largely due to a rapid loss of non-avid compared to avid total IgG; and (iv) both avid and non-avid total IgG in the absence of infection increased with age. Further studies are required to understand the functional differences between IgG1 and IgG3 in order to determine their contribution to the longevity of protective immunity to malaria. Measuring changes in antibody avidity may be a better approach of detecting affinity maturation compared to avidity index due to the differential expansion and contraction of high and low avidity total IgG.

## Introduction

IgG is an important component of immunity to malaria, with function defined by the specific recognition of antigens, and tropism of the constant region (Fc) for variant Fc receptors (1). Avidity, the sum of binding affinities between antibodies and antigenic epitopes, may reflect the functional quality of the antibody variable region (2, 3). IgG subclasses IgG1–IgG4 have differences in the Fc region that affect their affinity to variants of the Fcγ receptors, influencing their effector function, longevity, and ability to cross the placental barrier (4, 5). IgG subclass switching and affinity maturation (6, 7), are important components that dictate the quality of immunity to malaria (8–10).

Antibody levels and breadth of response to specific *P. falciparum* antigen targets generally diminish in the absence of re-infection, which is thought to contribute to loss of immunity (11–16). However, there are differences in the rate of decay of antibodies to different antigens (17, 18). Antibody responses to malaria are predominantly cytophilic (IgG1 and IgG3) and have been shown to mediate effector mechanisms that inhibit of parasite growth (19, 20), promote opsonic phagocytosis (21) and complement fixation (22, 23). Epidemiological studies have demonstrated associations between IgG1 and IgG3 targeting various *P. falciparum* antigens and different manifestations of immunity, including reductions in the risk of infection, parasite density, clinical disease, and disease severity (15, 20, 24, 25). In general, these associations were stronger for IgG3 compared to IgG1 (26–28). Furthermore, in-vitro functional assays have implicated interference by IgG2 and IgG4 in the opsonizing function of the cytophilic IgG1 and IgG3 antibodies in competition assays (29, 30).

Previous studies have shown differences in class switch bias profiles driven by different *P. falciparum* antigens (31–34). Other factors such as age and cumulative exposure are also thought to influence subclass switching (35). Therefore, the relative composition of the subclasses may influence the functional relevance of antibodies in antimalarial immunity.

Antibody avidity is a correlate for immune memory and protection in some infections (36–42). In malaria, studies have described an increase in affinity following resolution of clinical malaria (43), higher avidity in those with reduced risk of complicated malaria (44, 45), higher avidity in clinically immune compared to non-immune populations (43), and an association between higher avidity and reduced risk of placental malaria (10). Surprisingly, a prior study by our group demonstrated that avidity to the *P. falciparum* antigens AMA-1 and MSP1-19 was inversely related to transmission intensity at three sites in Uganda (46). This seemingly counterintuitive result prompted us to more measure avidity to broader array of antigens and to explicitly evaluate changes over time in individuals living in a setting of changing transmission intensity.

Few studies have combined the evaluation of IgG subclasses (IgG1-4) and avidity, and responses to only a limited number of *P. falciparum* antigens have been studied. We also do not fully understand the natural history of antibody waning in the absence of infection that may be important for naturally acquired immunity and its longevity. Understanding how naturally acquired immunity is maintained is important to identify populations at risk in settings with declining malaria transmission and to better inform malaria vaccine design.

In this study, we measured antibody levels to total IgG, IgG1–4 and avidity index (AI) to 18 *P. falciparum* blood stage antigens in the same individuals before and after decreases in malaria transmission (>90%) due to indoor residual spraying of insecticide (IRS). We compared the net changes in antibody responses during this period to gain a broader insight into how IgG subclasses and avidity index are acquired with age, and how they influence a reduction in IgG levels in the absence of infection.

## Methods

### Study Population

**Figure 1:**
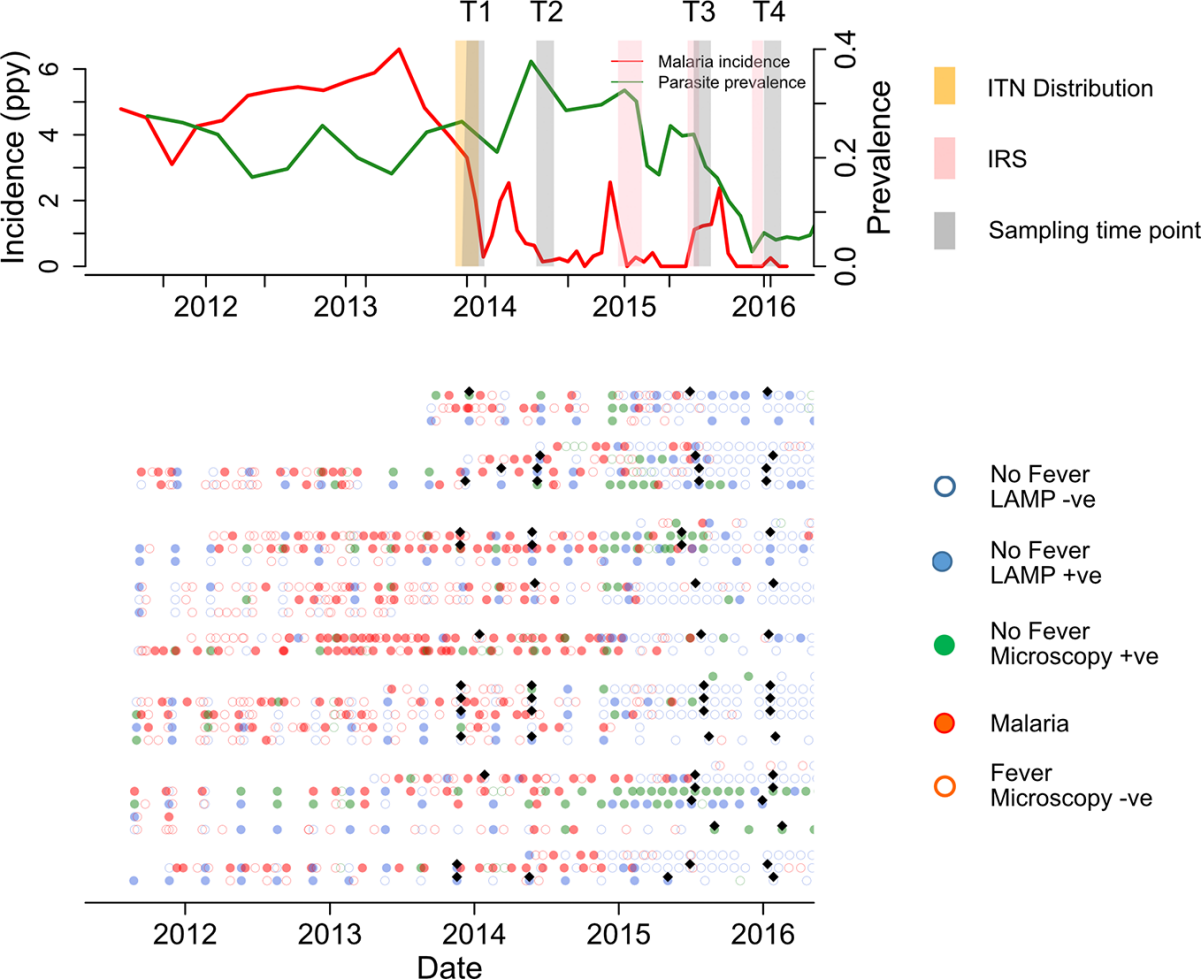
Summary of cohort at population and individual level. Upper panel shows malaria incidence (green line), parasite prevalence (red line), insecticide treated nets (ITN) distribution (yellow bar), and indoor residual spraying (IRS) spraying schedules (pink bars) in the full cohort. The lower panel represents the individual malaria status over 160 study sampling time points (black diamonds), at Time 1 (T1), T2, T3, and T4 (6 & 12 months before first IRS and 6 and 12 months after first IRS respectively). Participants were actively followed initially at 3 month intervals, which increased to every month in Oct 2016. Samples patent by microscopy and sub-patent by LAMP Infections are represented by green filled and blue filled circles respectively. Red filled, and red open circles represent malaria and non-malaria fever respectively. Green and blue open circles represent microscopy negative and loop mediated isothermal amplification (LAMP) negative, respectively.

Study participants were part of a cohort in Nagongera, eastern Uganda described in detail elsewhere (47). In 2011 Nagongera had one of the highest malaria burdens in the region with an entomological inoculation rate (EIR) of 215 infectious bites per person per year (48). Malaria control interventions included the use of insecticide treated nets (ITN), malaria case management with artemisinin-based therapies and intermittent presumptive treatment during pregnancy with sulfadoxine-pyrimethamine as per the Ugandan National Malaria Control Program policy. Between December 2014 and February 2015, IRS with the carbamate bendiocarb was introduced for the first time, followed by additional rounds ~ every 6 months thereafter. This intervention led to a dramatic decrease in the burden of malaria (**Figure 1**).

Plasma samples were selected from study participants (n=160) and classified into three age groups; 1–4 (n=40), 5–11 (n=92) and >18 years (n=28). Time points were selected at 12 and 6 months pre-IRS (T1 and T2, respectively), and at 6 and 12 months post-IRS (T3 and T4, respectively). Study participants were provided with ITN at enrollment and monitored for parasite prevalence and malaria incidence (47). Malaria cases were identified *via* passive surveillance. *P. falciparum* infection was identified by active surveillance at 1–3 monthly intervals using both microscopy and LAMP (49).

### Ethical Approval

Ethical approval was obtained from the Makerere University School of Medicine Research and Ethics Committee (REC REF 2011-203), the Uganda National Council for Science and Technology (HS 1074), the LSHTM ethics committee (reference # 6012), and the University of California, San Francisco Committee on Human Research (reference 027911).

Written informed consent was obtained from a parents or guardian of each child enrolled in the study and also from participating adults.

### *P. falciparum* Antigens

A total of 18 recombinant *P. falciparum* blood stage antigens were assessed in addition to tetanus toxoid (TT) (National Institute of Biological Service and Control) as a non-malaria control. The *P. falciparum* antigens broadly fell into three categories; (i) infected red blood cell, (ii) merozoite apical organelle, and (iii) merozoite surface antigen associated proteins. The recombinant antigens were expressed in *Escherichia coli* either as glutathione S-transferase (GST) fusion proteins or as histidine tagged constructs (**Supplementary Table 1**), with the exception of AMA1 (50) which was expressed in *Pichia pastoris* and Rh5.1 which was expressed in HEK293 mammalian cells (51). Antigen sequences were derived from the 3D7 isolate, with the exception of MSP2 (Dd2 and CH150/9 alleles) (52), MSP1-19 (FVO) (53) and GLURP RII (F32 allele) (54).

### Multiplex Bead Array Assay to Measure Total IgG and IgG1–4

Total IgG responses to 18 *P. falciparum* blood stage antigens and TT were assayed in plasma at 1/1,000 dilution using a multiplex bead array assay. Antigens were coupled to magnetic MagPlex microsphere beads (Luminex Corp, Austin, Texas) and assayed as previously described (55–58). Briefly, 50 µl of a pooled antigen-coupled bead suspension was added to each well of a 96-well plate, washed with PBS/Tween 20 and incubated with 50 µl of a 1/1,000 test plasma. A pool of hyperimmune Ugandan serum was used as a positive control. The plates were incubated 1.5 h at room temperature, washed (PBS/Tween 20) and goat anti-human IgG rPE labeled secondary antibody (Jackson Immuno Research Laboratories) was added and incubated for 1.5 h on a shaking platform, at room temperature. The plates were washed a final time, 1xPBS added to each well and the plates read on a MagPix machine (Luminex Corp, Austin, Texas). The results were expressed as median fluorescent intensity (MFI) and a standard curve based on the hyperimmune serum pool was included on each plate to normalize for plate to plate variations. The blank well MFI was deducted from each well to determine the net MFI.

To measure the IgG subclass responses, the total IgG assay described above was modified as follows. Test serum was used at 1/100 including a biotinylated anti-human IgG subclass (mouse anti-human IgG1: HP6069, IgG2: HP6002, IgG3: HP6050 IgG4: HP6023, Thermo fisher Scientific, UK) as the secondary antibody with a streptavidin-Phycoerythrin labeled tertiary component (Thermo fisher Scientific, UK), similar to what was previously described (59, 60). The mouse anti-human secondary antibodies were used at 1/400, 1/400, 1/1,000, and 1/400 for IgG1–4, respectively. The results were expressed as MFI was after subtracting the blank well.

### Multiplex Bead Array Assay to Measure IgG Avidity Index

To measure the avidity index, the total IgG assay was modified to include a Guanidine Hydrochloride (GuHCl) step to dissociate antibodies with low avidity. Briefly, 50µl GuHCl was added to one of the duplicate wells for each sample, after the plasma incubation step. PBS was added to the second well. After washing R-Phycoerythrin-conjugated AffiniPure F (ab’) 2 Goat anti-human IgG was added and processed as per the standard MagPix assay described above. The signal detected in the well treated with GuHCl represented high avidity antibodies. Avidity index was calculated as a percentage of the high avidity antibodies of the total antibody as shown below.

$$\text{Avidity}\;\text{Index}=\frac{\text{MFI}+\text{GuHCI}}{\text{MFI}+\text{PBS}}\times 100$$

### Statistical Analysis

The net changes in total IgG, IgG1–4, avidity index (AI) and the avid IgG levels (the antibody remaining bound after GuHCl treatment) between the peak and lowest malaria transmission were derived from a paired difference between log_10_ transformed MFI and AI at T2 and T4 respectively.

Associations between antibody levels (log_10_ transformed MFI) for total IgG, IgG1–4, avidity index and avid IgG with 90 days since last infection were assessed using the generalized estimation equation (GEE) to allow for repeated measures per individual including all 4 time points. Infections were defined by microscopy or loop mediated isothermal amplification (LAMP) for the smear negative slides.

Associations between total IgG, IgG1–4, avidity index and avidity index with age was assessed using a GEE model, adjusted for days since infection. Age groups 1–4 years was as the reference and compared to age groups 5–11 and 18 years.

Median (and interquartile range) differences for total IgG, IgG1–4, AI and avid IgG levels between age categories where compared in a one-way ANOVA, not assuming normal distribution (using nonparametric, Kruskal-Wallis test) with pairwise comparisons between all age groups.

Analysis were performed and figures generated using Stata version 14 (StataCorp LLC, USA), GraphPad Prism version 7 (Graphpad Software Inc, USA) and R-studio (RStudio, Inc, USA).

## Results

### Recombinant Protein Panel

The antigen panel used in the study was comprised of previously validated markers of seroincidence (34, 61, 62). All protein constructs were based on single alleles with the exception of MSP2 which was represented by two of the dimorphic types (Dd2 and CH150/9).Target sequences selected were anchored within conserved or semi-conserved regions of each protein (63) with the exception of AMA1 which was based on the full-length sequence with N- and C-terminal truncations (50) (**Supplementary Table S1**).

### Decline in IgG Levels Against *P. falciparum* Blood Stage Antigens With Decreasing Transmission

**Figure 2:**
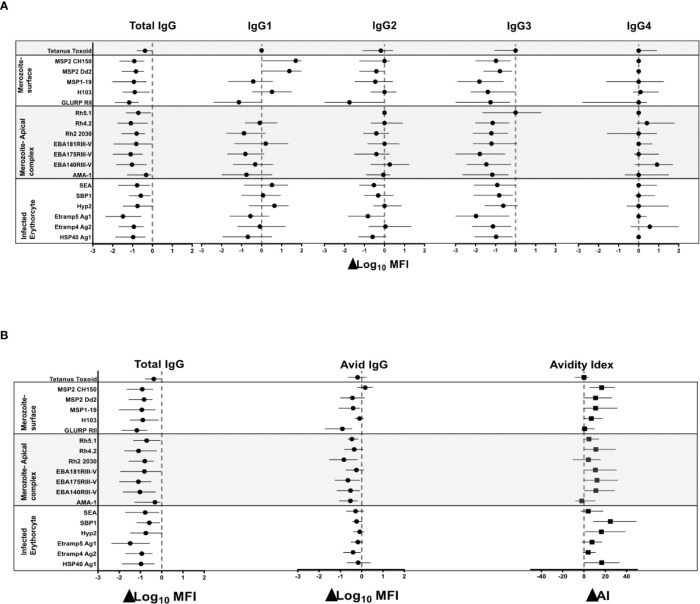
Net change in the median (IQR) of log_10_ MFI of total IgG, IgG1 – 4, avid IgG and avidity index between peak malaria transmission at T2 and near zero transmission at T4. **(A)** Total IgG and IgG1, IgG2, IgG3 and IgG4 **(B)** Total IgG and avid IgG and Avidity index. The broken red line denotes zero; no net change in responses. The black dot represents the median and the lines on either side the interquartile ranges.

IgG responses were detected to all 18 *P. falciparum* antigens evaluated, with median responses higher for IgG3 compared to IgG1 to all antigens except HSP40 ag1 and MSP1-19, for which responses were similar, and to AMA-1 and Rh2_2030 which were higher for IgG1 compared to IgG3 (**Supplementary Figure 1**). To determine if the reduction in transmission in Nagongera was associated with antibody responses, we assessed the difference in antibody levels between T2 (6 months pre-IRS; peak transmission) and T4 (12 months post IRS; lowest transmission) (**Figure 2** and **Table 1**). IgG levels (Log_10_MFI) were reduced for most of the *P. falciparum* antigens between the times of peak malaria transmission (T2) and lowest transmission (T4) (**Figure 2A**). A similar reduction was observed for almost all IgG3 responses, but this was not seen for IgG1, 2, or 4 responses. The reductions in the total IgG and IgG3 responses were similar for all the antigens tested. The largest reduction in total IgG was observed for Etramp5 ag1 and the smallest for AMA-1. The largest reduction in IgG3 was also observed for Etramp5 ag1 and the smallest for Rh5.1. IgG responses to tetanus toxoid, a non-malaria antigen control, did not change markedly between the two time points, consistent with the notion that the observed decreases were malaria specific. Taken together, these results showed that median total IgG levels declined dramatically between T2 and T4, mostly driven by reductions in IgG3 levels.

**Table 1:** Summary of population and malaria metrics.

Population and Malaria matrix	Age (years)	Time point			
		T1	T2	T3	T4
Population	1–4	40	40	40	38
	5–11	92	92	91	92
	>18	28	28	28	28
Proportion with at least 1 clinical malaria episode in the last 90 days (%)	1–4	61	73	39	0
	5–11	44	55	34	4
	>18	11	10	0	0
Proportion with at least 1 parasitemia episode in the last 90 days (%)	1–4	76	80	58	14
	5–11	95	89	85	45
	>18	58	70	45	14
Mean days since parasitemia	1–4	51	40	91	240
	5–11	9	23	50	146
	>18	77	84	186	322
Proportion of months free of parasite in the last 6 months	1–4	0.42	0.35	0.62	0.83
	5–11	0.27	0.25	0.41	0.62
	>18	0.48	0.53	0.71	0.85
Proportion of months free of parasite in the last 12 months	1–4	0.45	0.40	0.51	0.75
	5–11	0.29	0.29	0.37	0.51
	>18	0.49	0.51	0.64	0.78

Time points were relative to the first round of IRS. T1=12, T2=6 months before IRS. T3=6, T2=12 months after IRS. Malaria was defined by fever and positive blood smear microscopy. Parasitemia status was defined by microscopy or LAMP positive at monthly interval.

### Increase in Antibody Avidity Index to the *P. falciparum* Blood Stage Antigens With Decreasing Transmission

To determine if the reduction in malaria transmission was associated with antibody avidity, we measured differences in the avidity index and the avid total IgG (i.e. antibodies remaining after the GuHCl antibody dissociation step), between T2 and T4. The median avidity index increased for most of the *P. falciparum* antigens between T2 and T4, with the exception of GLURP RII, AMA-1, Rh5.1, and Etramp5 ag1 (**Figure 2B**). The largest net increase in avidity index was observed for antigens SBP1 and HSP40 ag1. The high avidity antibodies showed a minor decrease in the antibody responses for most antigens (**Figure 2B**). For GLURP RII, Rh2_2030, EBA181 RIII-V, EBA175 RIII-V, EBA140 RIII-V and AMA-1, the magnitude of the decrease in the high avidity antibodies was less than that of total IgG. There were no consistent patterns across the different categories of the *P. falciparum* antigens. Avidity index and avid pool to tetanus toxoid did not change between T2 and T4, as expected, again indicating that the observations were specific for *P. falciparum*. Since the difference between total IgG and the avid pool was an indirect measure of low avidity antibodies, these results indicated that increased avidity index was driven by a preferential loss of the pool of antibodies with lower avidity following marked reduction in transmission.

### The Decline in Total IgG Responses in the Absence of *P. falciparum* Infection Was Driven by Preferential Decay of IgG3

**Figure 3:**
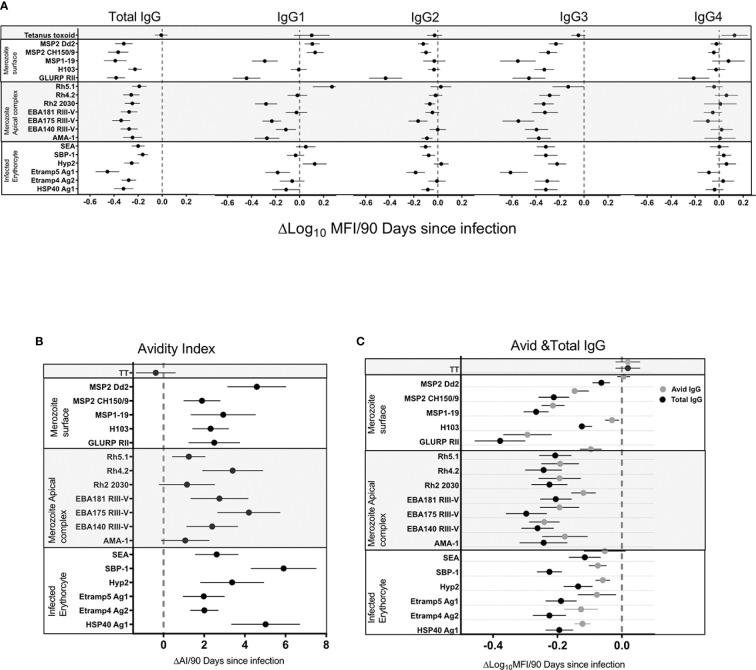
Association of total IgG, IgG1-4 **(A),** avidity index **(B)**, avid, and total IgG **(C)** 90 days following infection. The black filled circle represents the mean with error bars showing 95% confidence interval of the regression coefficient. Right of the zero red line is an increase and left is a decrease 90 days following infection. Overlap of the zero red line is a non-significant association.

We hypothesized that population level changes in antibody responses associated with a decrease in malaria transmission were due to a waning of antibodies in the absence of blood stage infection. To test this hypothesis and measure the rate of change, we took advantage of frequent active and continuous passive surveillance for *P. falciparum* infection to relate antibodies at all 4 time points (T1–T4) to the time since an individual’s last infection. Waning of antibodies was estimated using log-linear regression, i.e. assuming an exponential rate of decay and accounting for repeated measures using generalized estimating equations (GEE). Total IgG responses decreased following infection for all 18 *P. falciparum* antigens (**Figure 3A**). A significant decrease following infection was observed in IgG3 for all antigens (p=<0.0001) except Rh5.1 (p=ns). IgG1 responses were more heterogeneous, showing mostly non-significant decreased, unchanged, or increased trends following infection (**Figure 3A**). IgG2 responses showed little or no change following infection (**Supplementary Table 2**). IgG4 responses showed minimal reduction for all antigens except GLURP RII (p=0.001) (**Supplementary Table S2**). There was no change in the tetanus toxoid responses following *P. falciparum* infection, confirming that these results were malaria specific. Taken together, the results demonstrated that waning total IgG responses following infection were consistently driven by decreases in IgG3 and occasionally by decreases in IgG1 responses (**Supplementary Table S2**).

### Increased *P. falciparum* Specific Antibody Avidity Index Following Infection Was Due to a Preferential Loss of Low Avidity Antibodies

We hypothesized that the population level increase in antibody avidity index following a decrease in malaria transmission was due to the differential waning of total verses high avidity antibodies in the absence of blood stage infection. To test this hypothesis and measure the rate of decline in total verses avid antibodies, and the rate of change in the avidity index, we related antibodies and avidity index at all four time points (T1–T4) to the duration of time since an individual’s last infection. Changes were estimated using log-linear or linear regression for antibody levels and avidity index respectively, i.e. assuming an exponential rate of change. The IgG avidity index increased significantly (p=<0.0001) following infection for all antigens except AMA-1 (Confidence Interval (CI) −0.001–0.025), Rh2_2030 (CI -0.002-0.028) and Rh5.1 (CI 0.004-0.022) (**Figure 3B**) (p=ns). The largest increase in avidity index following infection was seen for HSP40 ag1 (p=<0.0001, CI 0.037–0.074), SBP1 (p=<0.0001, CI 0.017–0.083) and MSP2_Dd2 (p=0.0001, CI 0.034–0.066) (**Supplementary Table S3**). There was no noticeable trend associated with the antigen categories of merozoite surface, merozoite apical complex and infected erythrocytes. As expected, the avidity index of IgG against tetanus toxoid did not change following *P. falciparum* infection, again confirming the specificity of the responses to the malarial antigens. In order to determine the driver of the increased avidity index in the absence of infection, the rate of total IgG decay was compared to avid IgG. The rate of decay following infection was higher in total compared to the avid IgG pool for all 18 antigens (**Figure 3C**). Because the avid IgG pool is a component of total IgG, the relative difference in the rate of decay following infection can be attributed to the non-avid pool. Thus, a preferential decay of non-avid antibodies is a likely explanation for the observed increase in avidity index.

### The Rate of Antibody Decay Following Infection Declined With Age

**Figure 4:**
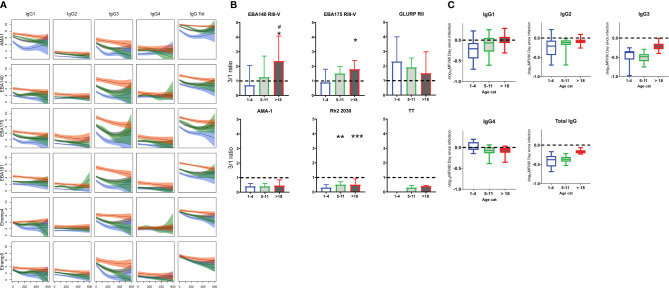
Changes in total IgG and subclasses 1–4 with age. **(A)** Representative plots of mean Log_10_ MFI against 90 days since last Infection, modeled using generalized additive models (GAMS), shaded areas represent 95% confidence interval. **(B)** Changes in IgG 3/1ratio with age. **(C)** Average rate (of 18 antigen) of decay following infection by age. Blue =1–4 years (N= 40), Green = 5–11 (N= 92) and Red = >18 years (N= 28). Gray = average (N=160) Ns, not significant, * = p= 0.05 – 0.01, **p = 0.009 – 0.0001 ***p > 0.00001.

Total IgG levels decayed faster in the younger (1–4 and 5–11 years) verses older (>18 years) age groups for the majority of the antigens (**Figures 4A, C**, and **Supplementary Figure 2**). The relative difference in the decay slopes was more pronounced in the first 200 days following infection, and in IgG3 compared to IgG1. There was little change in the rate of decay with age for IgG2 and 4 for most antigens (**Figures 4A, C**, and **Supplementary Figure 2**). The most pronounced changes across age category were observed for AMA-1, EBA175 RIII-V, EBA181 RIII-V, GLURP RII MSP2_Dd2, and MSP2_CH150/9. By contrast Etramp4 ag2, Etramp5 ag1 and HSP40 ag1 showed minimal changes across age. IgG1 and IgG3 had similar trends across the age categories for all the malaria antigens, with IgG3 showing more pronounced changes. The differences between ages were at maximum around 200 days following infection for total IgG, IgG1, and IgG3, driven by a rapid loss of antibodies associated with days since last infection in the 1–4 year age category compared to the older ages (5–11 and >18 years).

To determine changes in the relative proportion of IgG1 and IgG3 with age, a ratio of Log_10_MFI IgG3 to IgG1 was determined and compared across the three age categories at T4 (to represent the antibody profile in the relative absence of recent infection). A ratio below 1 implied a higher proportion of IgG1 and a ratio above 1 a higher proportion of IgG3. For the 1–4 year age category the median IgG3/IgG1 ratio was below 1 for all antigens except for GLURP RII (**Figure 4B** and **Supplementary Figure 3**). Median IgG3/IgG1 ratio increased for ages 5–11 and >18 years, approaching or exceeding 1 for most antigens except for AMA-1, Rh2_2030, HSP40 ag1 and Rh5.1, for which the median ratio remained below 1 in all age categories. By contrast, the IgG3/IgG1 ratio of tetanus toxoid remained below 1 for all age categories.

### High Avidity Antibodies Increased but the Avidity Index Decreased or Remained Unchanged With Age

**Figure 5:**
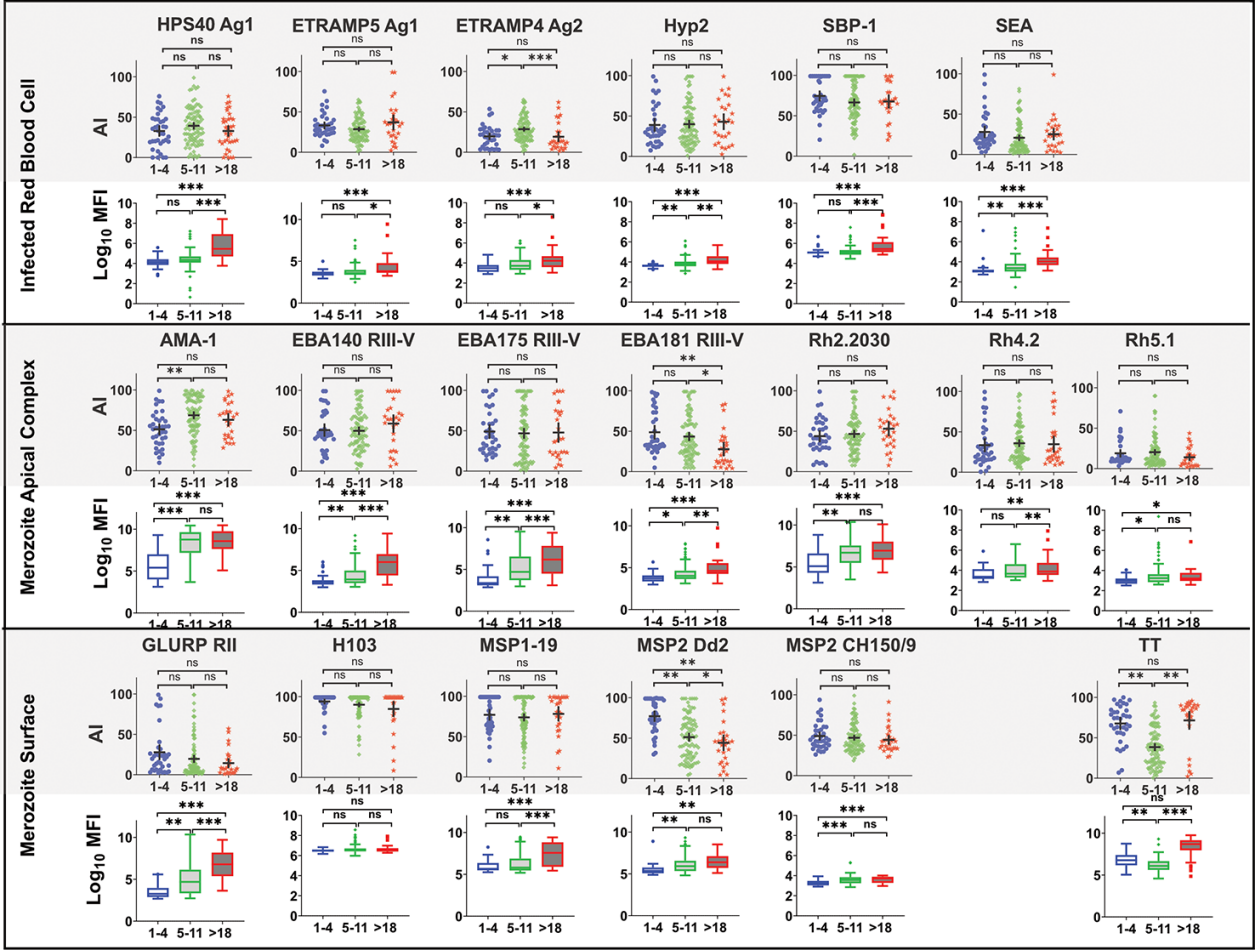
Comparison of Avidity index and high avidity antibodies across age category at T4. High avidity antibodies that remained binding after treatment with 2M GuHCl. Avidity index is the percentage of avid antibody of the total. Antibody was measure in a multiplex bead assay including 18 *P. falciparum* blood stage antigens and tetanus toxoid (TT). Age categories at T4 include 1–4 years (N= 40), 5–11 (N= 92) and >18 years (N= 28). Ns, not significant, * = p= 0.05 – 0.01, ** p = 0.009 – 0.0001 *** p>0.00001.

There was no significant association between age and avidity index for most of the antigens, except for Etramp4 ag2 (1–4 vs 5–11, p=0.007, 5–11 vs >18, p =0.012), EBA181 RIII-V (1–4 vs >18, p=0.006, 5–11 vs >18, p =0.013), MSP2 Dd2 (1–4 vs >18, p<0.0001, 5–11 vs >18, p<0.0001), and AMA-1(1–4 vs 5–11, p=0.0004), for which avidity index was higher for age categories 1–4 compared with 5–11 and with >18 years (**Figure 5**). By contrast, there was significant increase in high avidity antibodies across all age categories for all antigens except H103, particularly between age categories 5–11 and >18 years. There was a significant decrease in avidity index for TT between age categories 1–4 and 5–11 years, and an increase in the >18year age category. A similar pattern was observed for the high avidity antibodies. The restoration of both avidity index and high avidity antibodies may be attributed to a possible TT vaccination boost.

## Discussion

We measured responses to a panel of 18 *P. falciparum* blood stage antigens to gain broad insight into the dynamics of antibody levels and avidity when malaria transmission is reduced.

The majority of the targets used were based on the 3D7 allelic sequence, all the targets were based largely on conserved or semi-conserved targets. All of which had been previously evaluated as important markers of prior exposure to infection (61, 63). The exceptions to this were MSP2, AMA1, and GLURP RII. MSP2 falls into two dimorphic types (Type A and B) represented here by the CH150/9 (type A) and Dd2 (type B) (52). Despite the level of repetition within the MSP2 proteins studies have used a single allele of MSP2 in combination with other recombinant targets to describe immunity to infection (64), or have opted for representative sequences from both major dimorphic types as has been done for this study. Lastly, the MSP2-C1 blood stage vaccine is also based on a single representative type from each of the dimorphic classifications (3D7 and Fc27) (65, 66). Although the importance of polymorphism in AMA1 has been demonstrated in protective immunity (67–69) in terms of measuring exposure to infection extensive studies have demonstrated that a single allele was sufficient to adequately capture exposure to infection (62, 70, 71), including in the establishment of a WHO reference reagent for anti-malaria human serum (72). In addition, Greenhouse and colleagues previously demonstrated a highly correlative antibody response between the 3D7 and FVO alleles of AMA1 (73). With MSP1-19, there is a high degree of conservation within *P.falciparum* (74). Further to this, Hill et al demonstrated strain transcending immunity between 15 different parasite strains, including 3D7, in antibody based opsonization assays (75). Finally, although the GLURP RII antigen is anchored within the carboxy terminal repeat region (54) and as such there is a potential for avidity responses may vary between antigen types. However, it has been demonstrated in a number of studies to be an important marker of prior exposure to infection (56, 76).

Consistent with previous studies, we showed that total IgG increased with age (10), that it substantially declined when exposure decreased, that IgG1 and IgG3 were the dominant subclasses, and that there was antigen specific bias of dominance to these subclasses (20, 21, 25). In addition to previous findings, we showed that the decrease in total IgG was consistently driven by the decrease in IgG3 and occasionally by a decrease in IgG1. We also showed that the proportion of IgG3 relative to IgG1 maintained in the absence of infection increased with age, and that the rate of antibody decay following infection declined with age.

We validated previous findings that the antimalarial antibody avidity index increased following infection and was not consistently associated with age (39, 49). In addition to previous work (46), we showed that the increase in avidity index following infection was likely due to the more rapid loss of the low avidity vs. high avidity antibodies.

Differences in individual antibody avidity is likely to be on a continuous spectrum. Use of GuHCl at one concentration to define avidity results in dichotomization of the data. Therefore, though useful, the designation of “avid” and “non-avid” is an over-simplification. We postulate that the low avidity antibodies are produced mainly by short-lived plasma cells (SLPC), and high avidity antibodies by long lived plasma cells (LLPC). Infection drives expansion of the SLPC and non-avid antibodies, and the rapid contraction of SLPCs following infection leads to rapid preferential decay of the low avidity antibodies. Therefore, the increase in avidity index we observed may indicate affinity maturation per se and/or the relative contributions of SLPCs and LLPCs following infection. A study conducted in the setting of ongoing malaria transmission and infection in Nigeria found that antibody avidity was stable among most individuals over time (77); this finding is largely consistent with our proposed model. In future studies, the direct quantification of high avidity antibodies—not performed here—may be a better indicator of affinity maturation compared to the avidity index. For example, we demonstrated that high avidity antibodies increased with age despite the lack of an increase in the avidity index with age.

The implication of early acquisition of the predominantly IgG1 response we observed is not well understood, however there is increasing evidence to suggest that IgG3 may be more protective than other subtypes against malaria. A stronger association between IgG3 and a reduced risk of malaria compared to IgG1 was reported against PfRH5 and PfRipr, (27). In addition IgG3 C1q fixing antibodies to MSP2 and EBA175 RIII-V were associated with parasite clearance following treatment (78). IgG3 has greater activity in promoting complement fixation and opsonic phagocytosis, which are mechanisms that appear to contribute to protective immunity (21, 23, 79). The observation that IgG3 decayed at a faster rate than IgG1 in the absence of infection, and was significantly slower in adults compared to infants, implies a gradual accumulation of IgG3^+^ LLPC with age and exposure.

The finding of a shift in dominance from IgG1 to IgG3 with age is intriguing, especially when it is considered that subclass switching generally proceeds in a single direction (IgG3, to IgG1, to IgG2 and IgG4) due to the nature of the CH domains in the Fc region (5). Therefore, memory B cells (MBC) that eventually give rise to IgG3^+^ LLPC cannot be the cells that switched to IgG1. Rather, naïve B cells, IgG3^+^ MBC, or IgM^+^ MBC are likely to be the source of IgG3^+^LLPC that gradually accumulate with age (80). Some studies have not found a major effect of age and exposure on the IgG subclass responses to antigens (33) suggesting the nature of exposure or specific epidemiological conditions may influence this effect.

IgG3 has the shortest intrinsic half-life, at 7 days, compared to ~21 days for the other subclasses (81). This implies that maintaining a large pool of IgG3 in the absence of infection requires a larger pool of LLPC compared to IgG1. We observed a slower decay or increased half-life with age of IgG1 and IgG3, but with a shift in relative proportion from predominantly IgG1 to IgG3. This may imply gradual expansion of preferentially IgG3^+^ LLPC with age. Previous studies have indicated a requirement of high antibody titers to maintain immunity to malaria (15). As IgG3 is the predominant IgG for most of the malaria antigens we evaluated, a larger pool of IgG3^+^ LLPC may be required to maintain high titers compared to IgG1 in the absence of infection. While IgG3 is suspected to be functionally more potent compared to IgG1 (82), being the dominant subclass poses a limitation of poor maintenance of protective levels in the absence of infection. This limitation poses a big challenge to malaria vaccine development, especially in endemic areas where existing memory B cells are inclined to switch to IgG3^+^ LLPC. A recent analysis of RTS,S vaccine responses in children found IgG3 responses had a higher decay rate than IgG1 (83). Antigens such as AMA-1 and Rh2_2030 elicited a predominantly IgG1 response across the age groups. In addition, these antigens had a relatively lower decay rate following infection compared to antigens that were predominantly IgG3. This observation implies that some *P. falciparum* antigens may have inherent features that influence IgG class switch bias. Therefore, we need to better understand the functional potency of IgG1 verses IgG3 and antigen features that lead to biased class switching to inform malaria vaccine designs.

## Conclusions

In the setting of declining exposure to *P. falciparum*, IgG3 was the major driver of observed antibody decay rate and avidity index increased, likely due to preferential decay of the non-avid vs. avid antibodies. More direct quantification of avid antibodies, ascertainment of the cellular phenotype producing various antibodies over time, and functional studies of various aspects of antibody quality will be necessary to understand their protective roles in malarial immunity. This knowledge is critical for development and assessment of vaccine strategies that can improve longevity of malaria vaccine efficacy and in understanding the longevity of immunity to malaria as transmission declines.

## Data Availability Statement

The original contributions presented in the study are included in the article/**Supplementary Material**. Further inquiries can be directed to the corresponding author.

## Ethics Statement

The studies involving human participants were reviewed and approved by Makerere University School of Medicine Research and Ethics Committee (REC REF 2011-203), Uganda National Council for Science and Technology (HS 1074), LSHTM ethics committee (reference # 6012), University of California, San Francisco Committee on Human Research (reference 027911). Written informed consent to participate in this study was provided by the participants’ legal guardian/next of kin.

## Author Contributions

GD, MK, PR, BG, and CD designed the surveys. JR, EA, and JN conducted the surveys. IR, BG, and HM-K contributed to data analysis and interpretation. KT and JB supplied antigen constructs. KT generated and supplied protein reagents. IS and KT designed the assays. KT, IS, BG, IR, and CD designed the study. IS wrote the first draft of the manuscript with support from KT, BG, and CD. All authors contributed to the article and approved the submitted version.

## Funding

This study was supported by funds from the National Institutes of Health as part of the East African International Centers of Excellence in Malaria Research (ICMER) program (U19AI089674) and a Fogarty International Centre (FIC) Training in malaria research in Uganda award (D43TW7375). Additional support was provided by a Bloomsbury SET Award (Innovation Fellowship to KKAT; BSA14) under the UKRI Connecting Capabilities Fund (CCF). 

## Conflict of Interest

The authors declare that the research was conducted in the absence of any commercial or financial relationships that could be construed as a potential conflict of interest.

## Supplementary Material

Supplementary Figure 1 Dot Plot of log_10_MFI of Merozoite apical complex. Antibody levels were measured using a MagPix (Luminex) multiplex assay including 18 malaria blood stage antigens and TT. Each dot represents a single sample from 160 participants at 4 time points. The horizontal bar represents the median and 95% CI.

Supplementary Figure 2 Changes in total IgG and subclasses 1 – 4 with age. Total IgG and IgG1 – 4 for 19 *P falciparum* antigens and TT was measure in a multiplex bead array assay. Plots of mean Log_10_ MFI against days since last Infection, modeled using generalized additive models (GAMS), shaded areas represent 95% confidence interval.

Supplementary Figure 3 Changes in total IgG3/IgG1 ratio with age. Total IgG and IgG1 – 4 for 19 *P falciparum* antigens and TT was measure in a multiplex bead array assay. Blue =1-4 years (N= 40), Green = 5-11 (N= 92) and Red = >18 years (N= 28). Gray = average (N=160) Ns = not significant, * = p= 0.05 – 0.01, ** p = 0.009 – 0.0001 *** p>0.0000.

Supplementary Table 1 Summary of the *P.falciparum* Blood stage antigens.

Supplementary Table 2 Generalised estimating equation (GEE) summary statistical data for Total IgG and IgG subclasses (1–4) for days since parasitemia.

Supplementary Table 3 Generalised estimating equation (GEE) summary statistical data for the avidity index (AI) for days since parasitemia.

## References

[1] Wardemann H Murugan R From human antibody structure and function towards the design of a novel Plasmodium falciparum circumsporozoite protein malaria vaccine. Curr Opin Immunol (2018) 53:119–23. 10.1016/j.coi.2018.04.023 29751213

[2] Karush F Affinity and the Immune Response*. Ann N Y Acad Sci (1970) 169(1):56–64. 10.1111/j.1749-6632.1970.tb55970.x 4905692

[3] Tijani MK Reddy SB Langer C Beeson JG Wahlgren M Nwuba RI Factors influencing the induction of high affinity antibodies to Plasmodium falciparum merozoite antigens and how affinity changes over time. Sci Rep (2018) 8(1):9026–6. 10.1038/s41598-018-27361-w PMC5998021 29899351

[4] Vidarsson G Dekkers G Rispens T IgG Subclasses and Allotypes: From Structure to Effector Functions. Front Immunol (2014) 5:520. 10.3389/fimmu.2014.00520 25368619 PMC4202688

[5] Valenzuela NM Schaub S The Biology of Igg Subclasses and Their Clinical Relevance to Transplantation. Transplantation (2018) 102(1S Suppl 1):S7–13. 10.1097/TP.0000000000001816 29266057

[6] Gatto D Brink R The germinal center reaction. J Allergy Clin Immunol (2010) 126(5):898–907; quiz 908–9. 10.1016/j.jaci.2010.09.007 21050940

[7] DeFranco AL The germinal center antibody response in health and disease. F1000Research (2016) 5 Suppl 1. 10.12688/f1000research.7717.1 PMC4882753 27303636

[8] Cohen S McGREGOR IA Carrington S Gamma-globulin and acquired immunity to human malaria. Nature (1961) 192:733–7. 10.1038/192733a0 13880318

[9] Cohen S Butcher GA Properties of protective malarial antibody. Immunology (1970) 19(2):369–83. PMC1455757 4990404

[10] Tutterrow YL Salanti A Avril M Smith JD Pagano IS Ako S High Avidity Antibodies to Full-Length VAR2CSA Correlate with Absence of Placental Malaria. PloS One (2012) 7(6):e40049. 10.1371/journal.pone.0040049 22761948 PMC3383675

[11] Achtman AH Bull PC Stephens R Langhorne J Longevity of the immune response and memory to blood-stage malaria infection. Curr Top Microbiol Immunol (2005) 297:71–102. 10.1007/3-540-29967-X_3 16265903

[12] Kinyanjui SM Conway DJ Lanar DE Marsh K IgG antibody responses to Plasmodium falciparum merozoite antigens in Kenyan children have a short half-life. Malar J (2007) 6(1):82. 10.1186/1475-2875-6-82 17598897 PMC1920526

[13] Akpogheneta OJ Duah NO Tetteh KKA Dunyo S Lanar DE Pinder M Duration of Naturally Acquired Antibody Responses to Blood-Stage Plasmodium falciparum Is Age Dependent and Antigen Specific. Infect Immun (2008) 76(4):1748–55. 10.1128/IAI.01333-07 PMC2292892 18212081

[14] Alonso PL Sacarlal J Aponte JJ Leach A Macete E Aide P Duration of protection with RTS,S/AS02A malaria vaccine in prevention of Plasmodium falciparum disease in Mozambican children: single-blind extended follow-up of a randomised controlled trial. Lancet (2005) 366(9502):2012–8. 10.1016/S0140-6736(05)67669-6 16338450

[15] Osier FHA Fegan G Polley SD Murungi L Verra F Tetteh KKA Breadth and Magnitude of Antibody Responses to Multiple Plasmodium falciparum Merozoite Antigens Are Associated with Protection from Clinical Malaria. Infect Immun (2008) 76(5):2240–8. 10.1128/IAI.01585-07 PMC2346713 18316390

[16] Ferreira MU Kimura EAS Katzin AM Santos-Neto LL Ferrari JO Villalobos JM The IgG-subclass distribution of naturally acquired antibodies to Plasmodium falciparum, in relation to malaria exposure and severity. Ann Trop Med Parasitol (1998) 92(3):245–56. 10.1080/00034983.1998.11813287 9713539

[17] Mugyenyi CK Elliott SR Yap XZ Feng G Boeuf P Fegan G Declining Malaria Transmission Differentially Impacts the Maintenance of Humoral Immunity to Plasmodium falciparum in Children. J Infect Dis (2017) 216(7):887–98. 10.1093/infdis/jix370 PMC5853596 28973483

[18] Fowkes FJ McGready R Cross NJ Hommel M Simpson JA Elliott SR New Insights into Acquisition, Boosting, and Longevity of Immunity to Malaria in Pregnant Women. J Infect Dis (2012) 206(10):1612–21. 10.1093/infdis/jis566 PMC3475637 22966126

[19] White MT Bejon P Olotu A Griffin JT Bojang K Lusingu J A combined analysis of immunogenicity, antibody kinetics and vaccine efficacy from phase 2 trials of the RTS,S malaria vaccine. BMC Med (2014) 12(1):117. 10.1186/s12916-014-0117-2 25012228 PMC4227280

[20] Biryukov S Angov E Landmesser ME Spring MD Ockenhouse CF Stoute JA Complement and Antibody-mediated Enhancement of Red Blood Cell Invasion and Growth of Malaria Parasites. EBioMedicine (2016) 9:207–16. 10.1016/j.ebiom.2016.05.015 PMC4972486 27333049

[21] Osier FH Feng G Boyle MJ Langer C Zhou J Richards JS Opsonic phagocytosis of Plasmodium falciparum merozoites: mechanism in human immunity and a correlate of protection against malaria. BMC Med (2014) 12:108. 10.1186/1741-7015-12-108 24980799 PMC4098671

[22] Behet MC Kurtovic L van Gemert G-J Haukes CM Siebelink-Stoter R Graumans W The complement system contributes to functional antibody-mediated responses induced by immunization with Plasmodium falciparum malaria sporozoites. Infect Immun (2018) 86(7):e00920–17. 10.1128/IAI.00920-17 PMC6013677 29735521

[23] Boyle MJ Reiling L Feng G Langer C Osier FH Aspeling-Jones H Human antibodies fix complement to inhibit Plasmodium falciparum invasion of erythrocytes and are associated with protection against malaria. Immunity (2015) 42(3):580–90. 10.1016/j.immuni.2015.02.012 PMC4372259 25786180

[24] Cherif MK Ouédraogo O Sanou GS Diarra A Ouédraogo A Tiono A Antibody responses to P. falciparum blood stage antigens and incidence of clinical malaria in children living in endemic area in Burkina Faso. BMC Res Notes (2017) 10(1):472. 10.1186/s13104-017-2772-9 28886727 PMC5591548

[25] Saavedra-Langer R Marapara J Valle-Campos A Durand S Vásquez-Chasnamote ME Silva H IgG subclass responses to excreted-secreted antigens of Plasmodium falciparum in a low-transmission malaria area of the Peruvian Amazon. Malar J (2018) 17(1):328. 10.1186/s12936-018-2471-6 30200987 PMC6131892

[26] Dobaño C Santano R Vidal M Jiménez A Jairoce C Ubillos I Differential Patterns of IgG Subclass Responses to Plasmodium falciparum Antigens in Relation to Malaria Protection and RTS,S Vaccination. Front Immunol (2019) 10:439. 10.3389/fimmu.2019.00439 30930896 PMC6428712

[27] Weaver R Reiling L Feng G Drew DR Mueller I Siba PM The association between naturally acquired IgG subclass specific antibodies to the PfRH5 invasion complex and protection from Plasmodium falciparum malaria. Sci Rep (2016) 08 6:33094. 10.1038/srep33094 PMC5015043 27604417

[28] Oeuvray C Theisen M Rogier C Trape JF Jepsen S Druilhe P Cytophilic immunoglobulin responses to Plasmodium falciparum glutamate-rich protein are correlated with protection against clinical malaria in Dielmo, Senegal. Infect Immun (2000) 68(5):2617–20. 10.1128/IAI.68.5.2617-2620.2000 PMC97467 10768952

[29] Groux H Gysin J Opsonization as an effector mechanism in human protection against asexual blood stages of Plasmodium falciparum: Functional role of IgG subclasses. Res Immunol (1990) 141(5):529–42. 10.1016/0923-2494(90)90021-P 1704637

[30] Chaudhury S Regules JA Darko CA Dutta S Wallqvist A Waters NC Delayed fractional dose regimen of the RTS,S/AS01 malaria vaccine candidate enhances an IgG4 response that inhibits serum opsonophagocytosis. Sci Rep (2017) 7(1):7998. 10.1038/s41598-017-08526-5 28801554 PMC5554171

[31] Taylor RR Allen SJ Greenwood BM Riley EM IgG3 antibodies to Plasmodium falciparum merozoite surface protein 2 (MSP2): increasing prevalence with age and association with clinical immunity to malaria. Am J Trop Med Hyg (1998) 58(4):406–13. 10.4269/ajtmh.1998.58.406 9574783

[32] Metzger WG Okenu DMN Cavanagh DR Robinson JV Bojang KA Weiss HA Serum IgG3 to the Plasmodium falciparum merozoite surface protein 2 is strongly associated with a reduced prospective risk of malaria. Parasite Immunol (2003) 25(6):307–12. 10.1046/j.1365-3024.2003.00636.x 14507328

[33] Stanisic DI Richards JS McCallum FJ Michon P King CL Schoepflin S Immunoglobulin G subclass-specific responses against Plasmodium falciparum merozoite antigens are associated with control of parasitemia and protection from symptomatic illness. Infect Immun (2009) 77(3):1165–74. 10.1128/IAI.01129-08 PMC2643653 19139189

[34] Richards JS Stanisic DI Fowkes FJI Tavul L Dabod E Thompson JK Association between naturally acquired antibodies to erythrocyte-binding antigens of Plasmodium falciparum and protection from malaria and high-density parasitemia. Clin Infect Dis Off Publ Infect Dis Soc Am (2010) 51(8):e50–60. 10.1086/656413 20843207

[35] Tongren JE Drakeley CJ McDonald SLR Reyburn HG Manjurano A Nkya WMM Target antigen, age, and duration of antigen exposure independently regulate immunoglobulin G subclass switching in malaria. Infect Immun (2006) 74(1):257–64. 10.1128/IAI.74.1.257-264.2006 PMC1346665 16368979

[36] Hedman K Rousseau SA Measurement of avidity of specific IgG for verification of recent primary rubella. J Med Virol (1989) 27(4):288–92. 10.1002/jmv.1890270406 2723615

[37] Pour Abolghasem S Bonyadi MR Babaloo Z Porhasan A Nagili B Gardashkhani OA IgG avidity test for the diagnosis of acute Toxoplasma gondii infection in early pregnancy. Iran J Immunol IJI (2011) 8(4):251–5. 10.22034/iji.2011.17033 22201623

[38] Schlesinger Y Granoff DM Avidity and bactericidal activity of antibody elicited by different haemophilus influenzae type b conjugate vaccines. The Vaccine Study Group. JAMA (1992) 267(11):1489–94. 10.1001/jama.267.11.1489 1538539

[39] Cremers AJH Lut J Hermans PWM Meis JF de Jonge MI Ferwerda G Avidity of Antibodies against Infecting Pneumococcal Serotypes Increases with Age and Severity of Disease. Clin Vaccine Immunol (2014) 21(6):904–7. 10.1128/CVI.00147-14 PMC4054233 24759650

[40] Goldblatt D Vaz AR Miller E Antibody avidity as a surrogate marker of successful priming by Haemophilus influenzae type b conjugate vaccines following infant immunization. J Infect Dis (1998) 177(4):1112–5. 10.1086/517407 9534995

[41] Park DW Nam M-H Kim JY Kim HJ Sohn JW Cho Y Mumps outbreak in a highly vaccinated school population: assessment of secondary vaccine failure using IgG avidity measurements. Vaccine (2007) 25(24):4665–70. 10.1016/j.vaccine.2007.04.013 17498856

[42] Sanz-Moreno JC Limia-Sánchez A García-Comas L Mosquera-Gutiérrez MM Echevarria-Mayo JE Castellanos-Nadal A Detection of secondary mumps vaccine failure by means of avidity testing for specific immunoglobulin G. Vaccine (2005) 23(41):4921–5. 10.1016/j.vaccine.2005.05.018 15996797

[43] Ferreira MU Kimura EA De Souza JM Katzin AM The isotype composition and avidity of naturally acquired anti-Plasmodium falciparum antibodies: differential patterns in clinically immune Africans and Amazonian patients. Am J Trop Med Hyg (1996) 55(3):315–23. 10.4269/ajtmh.1996.55.315 8842122

[44] Leoratti FM Durlacher RR Lacerda MV Alecrim MG Ferreira AW Sanchez MC Pattern of humoral immune response to Plasmodium falciparum blood stages in individuals presenting different clinical expressions of malaria. Malar J (2008) 7:186. 10.1186/1475-2875-7-186 18816374 PMC2559846

[45] Reddy SB Anders RF Beeson JG Färnert A Kironde F Berenzon SK High affinity antibodies to Plasmodium falciparum merozoite antigens are associated with protection from malaria. PloS One (2012) 7(2):e32242. 10.1371/journal.pone.0032242 22363818 PMC3283742

[46] Ssewanyana I Arinaitwe E Nankabirwa JI Yeka A Sullivan R Kamya MR Avidity of anti-malarial antibodies inversely related to transmission intensity at three sites in Uganda. Malar J (2017) 16(1):67. 10.1186/s12936-017-1721-3 28183299 PMC5301436

[47] Kamya MR Arinaitwe E Wanzira H Katureebe A Barusya C Kigozi SP Malaria Transmission, Infection, and Disease at Three Sites with Varied Transmission Intensity in Uganda: Implications for Malaria Control. Am J Trop Med Hyg (2015) 92(5):903–12. 10.4269/ajtmh.14-0312 PMC4426576 25778501

[48] Kilama M Smith DL Hutchinson R Kigozi R Yeka A Lavoy G Estimating the annual entomological inoculation rate for Plasmodium falciparum transmitted by Anopheles gambiae s.l. using three sampling methods in three sites in Uganda. Malar J (2014) 13:111. 10.1186/1475-2875-13-111 24656206 PMC4001112

[49] Nankabirwa JI Briggs J Rek J Arinaitwe E Nayebare P Katrak S Persistent Parasitemia Despite Dramatic Reduction in Malaria Incidence After 3 Rounds of Indoor Residual Spraying in Tororo, Uganda. J Infect Dis (2019) 219(7):1104–11. 10.1093/infdis/jiy628 PMC6420168 30383230

[50] Collins CR Withers-Martinez C Hackett F Blackman MJ An Inhibitory Antibody Blocks Interactions between Components of the Malarial Invasion Machinery. PloS Pathog (2009) 5(1):e1000273. 10.1371/journal.ppat.1000273 19165323 PMC2621342

[51] Jin J Tarrant RD Bolam EJ Angell-Manning P Soegaard M Pattinson DJ Production, quality control, stability, and potency of cGMP-produced Plasmodium falciparum RH5.1 protein vaccine expressed in Drosophila S2 cells. NPJ Vaccines (2018) 3:32. 10.1038/s41541-018-0071-7 30131879 PMC6098134

[52] Taylor RR Smith DB Robinson VJ McBride JS Riley EM Human antibody response to Plasmodium falciparum merozoite surface protein 2 is serogroup specific and predominantly of the immunoglobulin G3 subclass. Infect Immun (1995) 63(11):4382–8. 10.1128/IAI.63.11.4382-4388.1995 PMC173623 7591074

[53] Burghaus PA Holder AA Expression of the 19-kilodalton carboxy-terminal fragment of the Plasmodium falciparum merozoite surface protein-1 in Escherichia coli as a correctly folded protein. Mol Biochem Parasitol (1994) 64(1):165–9. 10.1016/0166-6851(94)90144-9 8078519

[54] Theisen M Vuust J Gottschau A Jepsen S Høgh B Antigenicity and immunogenicity of recombinant glutamate-rich protein of Plasmodium falciparum expressed in Escherichia coli. Clin Diagn Lab Immunol (1995) 2(1):30–4. 10.1128/CDLI.2.1.30-34.1995 PMC170096 7719909

[55] Wu L Hall T Ssewanyana I Oulton T Patterson C Vasileva H Optimisation and standardisation of a multiplex immunoassay of diverse Plasmodium falciparum antigens to assess changes in malaria transmission using sero-epidemiology. Wellcome Open Res (2019) 4:26. 10.12688/wellcomeopenres.14950.1 32518839 PMC7255915

[56] Kana IH Singh SK Garcia-Senosiain A Dodoo D Singh S Adu B Breadth of functional antibodies is associated with Plasmodium falciparum merozoite phagocytosis and protection against febrile malaria. J Infect Dis (2019) 220(2):275–84. 10.1093/infdis/jiz088/5367431 30820557

[57] Varela ML Mbengue B Basse A Loucoubar C Vigan-Womas I Dièye A Optimization of a magnetic bead-based assay (MAGPIX®-Luminex) for immune surveillance of exposure to malaria using multiple Plasmodium antigens and sera from different endemic settings. Malar J (2018) 17(1):324. 10.1186/s12936-018-2465-4 30189885 PMC6127931

[58] Valmaseda A Macete E Nhabomba A Guinovart C Aide P Bardají A Identifying Immune Correlates of Protection Against Plasmodium falciparum Through a Novel Approach to Account for Heterogeneity in Malaria Exposure. Clin Infect Dis (2018) 66(4):586–93. 10.1093/cid/cix837 29401272

[59] Cham GK Kurtis J Lusingu J Theander TG Jensen AT Turner L. A semi-automated multiplex high-throughput assay for measuring IgG antibodies against Plasmodium falciparum erythrocyte membrane protein 1 (PfEMP1) domains in small volumes of plasma. Malar J (2008) 7(1):108. 10.1186/1475-2875-7-108 18549480 PMC2435541

[60] Fernandez-Becerra C Sanz S Brucet M Stanisic DI Alves FP Camargo EP Naturally-acquired humoral immune responses against the N- and C-termini of the Plasmodium vivax MSP1 protein in endemic regions of Brazil and Papua New Guinea using a multiplex assay. Malar J (2010) 9(1):29. 10.1186/1475-2875-9-29 20092651 PMC2835717

[61] Gray JC Corran PH Mangia E Gaunt MW Li Q Tetteh KKA Profiling the antibody immune response against blood stage malaria vaccine candidates. Clin Chem (2007) 53(7):1244–53. 10.1373/clinchem.2006.081695 17510307

[62] Helb DA Tetteh KKA Felgner PL Skinner J Hubbard A Arinaitwe E Novel serologic biomarkers provide accurate estimates of recent Plasmodium falciparum exposure for individuals and communities. Proc Natl Acad Sci U S A (2015) 112(32):E4438–47. 10.1073/pnas.1501705112 PMC4538641 26216993

[63] Richards JS Arumugam TU Reiling L Healer J Hodder AN Fowkes FJI Identification and prioritization of merozoite antigens as targets of protective human immunity to Plasmodium falciparum malaria for vaccine and biomarker development. J Immunol Baltim Md (1950) 2013 191(2):795–809. 10.4049/jimmunol.1300778 PMC3702023 23776179

[64] Mbengue B Fall MM Varela M-L Loucoubar C Joos C Fall B Analysis of antibody responses to selected Plasmodium falciparum merozoite surface antigens in mild and cerebral malaria and associations with clinical outcomes. Clin Exp Immunol (2019) 196(1):86–96. 10.1111/cei.13254 30580455 PMC6422657

[65] McCarthy JS Marjason J Elliott S Fahey P Bang G Malkin E A phase 1 trial of MSP2-C1, a blood-stage malaria vaccine containing 2 isoforms of MSP2 formulated with Montanide® ISA 720. PloS One (2011) 6(9):e24413. 10.1371/journal.pone.0024413 21949716 PMC3176224

[66] Feng G Boyle MJ Cross N Chan J-A Reiling L Osier F Human Immunization With a Polymorphic Malaria Vaccine Candidate Induced Antibodies to Conserved Epitopes That Promote Functional Antibodies to Multiple Parasite Strains. J Infect Dis (2018) 218(1):35–43. 10.1093/infdis/jiy170 29584918 PMC6904323

[67] Ouattara A Niangaly A Adams M Coulibaly D Kone AK Traore K Epitope-based sieve analysis of Plasmodium falciparum sequences from a FMP2.1/AS02A vaccine trial is consistent with differential vaccine efficacy against immunologically relevant AMA1 variants. Vaccine (2020) 38(35):5700–6. 10.1016/j.vaccine.2020.06.035 PMC7375901 32571720

[68] Berry AA Gottlieb ER Kouriba B Diarra I Thera MA Dutta S Immunoglobulin G subclass and antibody avidity responses in Malian children immunized with Plasmodium falciparum apical membrane antigen 1 vaccine candidate FMP2.1/AS02A. Malar J (2019) 18(1):13. 10.1186/s12936-019-2637-x 30658710 PMC6339315

[69] Kamuyu G Tuju J Kimathi R Mwai K Mburu J Kibinge N KILchip v1.0: A Novel Plasmodium falciparum Merozoite Protein Microarray to Facilitate Malaria Vaccine Candidate Prioritization. Front Immunol (2018) 9:2866. 10.3389/fimmu.2018.02866 30619257 PMC6298441

[70] Assefa A Ali Ahmed A Deressa W Sime H Mohammed H Kebede A Multiplex serology demonstrate cumulative prevalence and spatial distribution of malaria in Ethiopia. Malar J (2019) 18(1):246. 10.1186/s12936-019-2874-z 31331340 PMC6647069

[71] Yman V White MT Asghar M Sundling C Sondén K Draper SJ Antibody responses to merozoite antigens after natural Plasmodium falciparum infection: kinetics and longevity in absence of re-exposure. BMC Med (2019) 17(1):22. 10.1186/s12916-019-1255-3 30696449 PMC6352425

[72] Bryan D Silva N Rigsby P Dougall T Corran P Bowyer PW The establishment of a WHO Reference Reagent for anti-malaria (Plasmodium falciparum) human serum. Malar J (2017) 05 16(1):314. 10.1186/s12936-017-1958-x PMC5545088 28779755

[73] Greenhouse B Ho B Hubbard A Njama-Meya D Narum DL Lanar DE Antibodies to Plasmodium falciparum antigens predict a higher risk of malaria but protection from symptoms once parasitemic. J Infect Dis (2011) 204(1):19–26. 10.1093/infdis/jir223 21628654 PMC3105040

[74] Miller LH Roberts T Shahabuddin M McCutchan TF Analysis of sequence diversity in the Plasmodium falciparum merozoite surface protein-1 (MSP-1). Mol Biochem Parasitol (1993) 59(1):1–14. 10.1016/0166-6851(93)90002-F 8515771

[75] Hill DL Wilson DW Sampaio NG Eriksson EM Ryg-Cornejo V Harrison GLA Merozoite Antigens of Plasmodium falciparum Elicit Strain-Transcending Opsonizing Immunity. Infect Immun (2016) 84(8):2175–84. 10.1128/IAI.00145-16 PMC4962632 27185785

[76] Adamou R Dechavanne C Sadissou I d’Almeida T Bouraima A Sonon P Plasmodium falciparum merozoite surface antigen-specific cytophilic IgG and control of malaria infection in a Beninese birth cohort. Malar J (2019) 18(1):194. 10.1186/s12936-019-2831-x 31185998 PMC6560827

[77] Tijani MK Babalola OA Odaibo AB Anumudu CI Asinobi AO Morenikeji OA Acquisition, maintenance and adaptation of invasion inhibitory antibodies against Plasmodium falciparum invasion ligands involved in immune evasion. PloS One (2017) 12(8):e0182187. 10.1371/journal.pone.0182187 28787025 PMC5546579

[78] O’Flaherty K Ataíde R Zaloumis SG Ashley EA Powell R Feng G Contribution of Functional Antimalarial Immunity to Measures of Parasite Clearance in Therapeutic Efficacy Studies of Artemisinin Derivatives. J Infect Dis (2019) 220(7):1178–87. 10.1093/infdis/jiz247 PMC6735958 31075171

[79] Joos C Marrama L Polson HEJ Corre S Diatta A-M Diouf B Clinical protection from falciparum malaria correlates with neutrophil respiratory bursts induced by merozoites opsonized with human serum antibodies. PloS One (2010) 5(3):e9871. 10.1371/journal.pone.0009871 20360847 PMC2845614

[80] Krishnamurty AT Thouvenel CD Portugal S Keitany GJ Kim KS Holder A Somatically Hypermutated Plasmodium-Specific IgM(+) Memory B Cells Are Rapid, Plastic, Early Responders upon Malaria Rechallenge. Immunity (2016) 45(2):402–14. 10.1016/j.immuni.2016.06.014 PMC5118370 27473412

[81] Mankarious S Lee M Fischer S Pyun KH Ochs HD Oxelius VA The half-lives of IgG subclasses and specific antibodies in patients with primary immunodeficiency who are receiving intravenously administered immunoglobulin. J Lab Clin Med (1988) 112(5):634–40. 3183495

[82] Irani V Guy AJ Andrew D Beeson JG Ramsland PA Richards JS Molecular properties of human IgG subclasses and their implications for designing therapeutic monoclonal antibodies against infectious diseases. Mol Immunol (2015) 67(2 Pt A):171–82. 10.1016/j.molimm.2015.03.255 25900877

[83] Kurtovic L Agius PA Feng G Drew DR Ubillos I Sacarlal J Induction and decay of functional complement-fixing antibodies by the RTS,S malaria vaccine in children, and a negative impact of malaria exposure. BMC Med (2019) 17(1):45. 10.1186/s12916-019-1277-x 30798787 PMC6388494

