# Supplementary figures and images for: Impact of a Rapid Decline in Malaria Transmission on Antimalarial IgG Subclasses and Avidity
Source: Front Immunol. 2021 Jan 27;11:576663. doi: 10.3389/fimmu.2020.576663 (PMC7873448; doi:10.3389/fimmu.2020.576663)

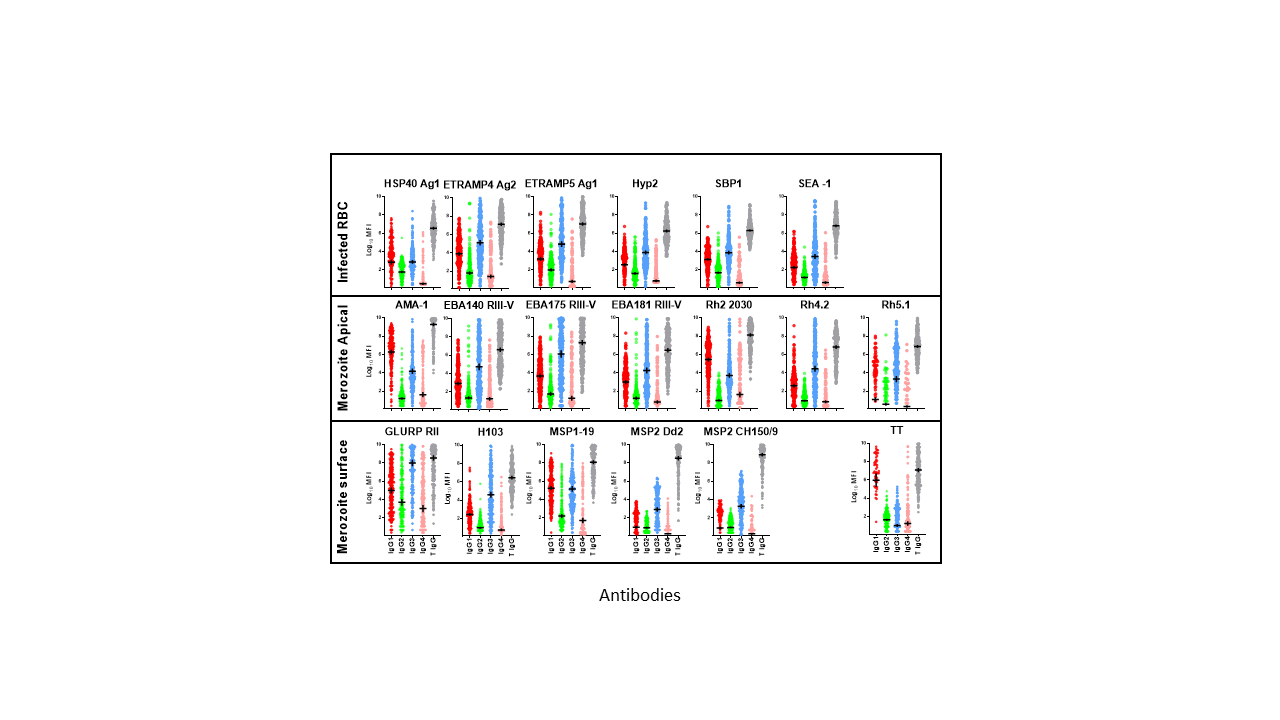

Supplement: Supplementary Figure 1 — Dot Plot of log10MFI of Merozoite apical complex. Antibody levels were measured using a MagPix (Luminex) multiplex assay including 18 malaria blood stage antigens and TT. Each dot represents a single sample from 160 participants at 4 time points. The horizontal bar represents the median and 95% CI. [file Image_1.tif]

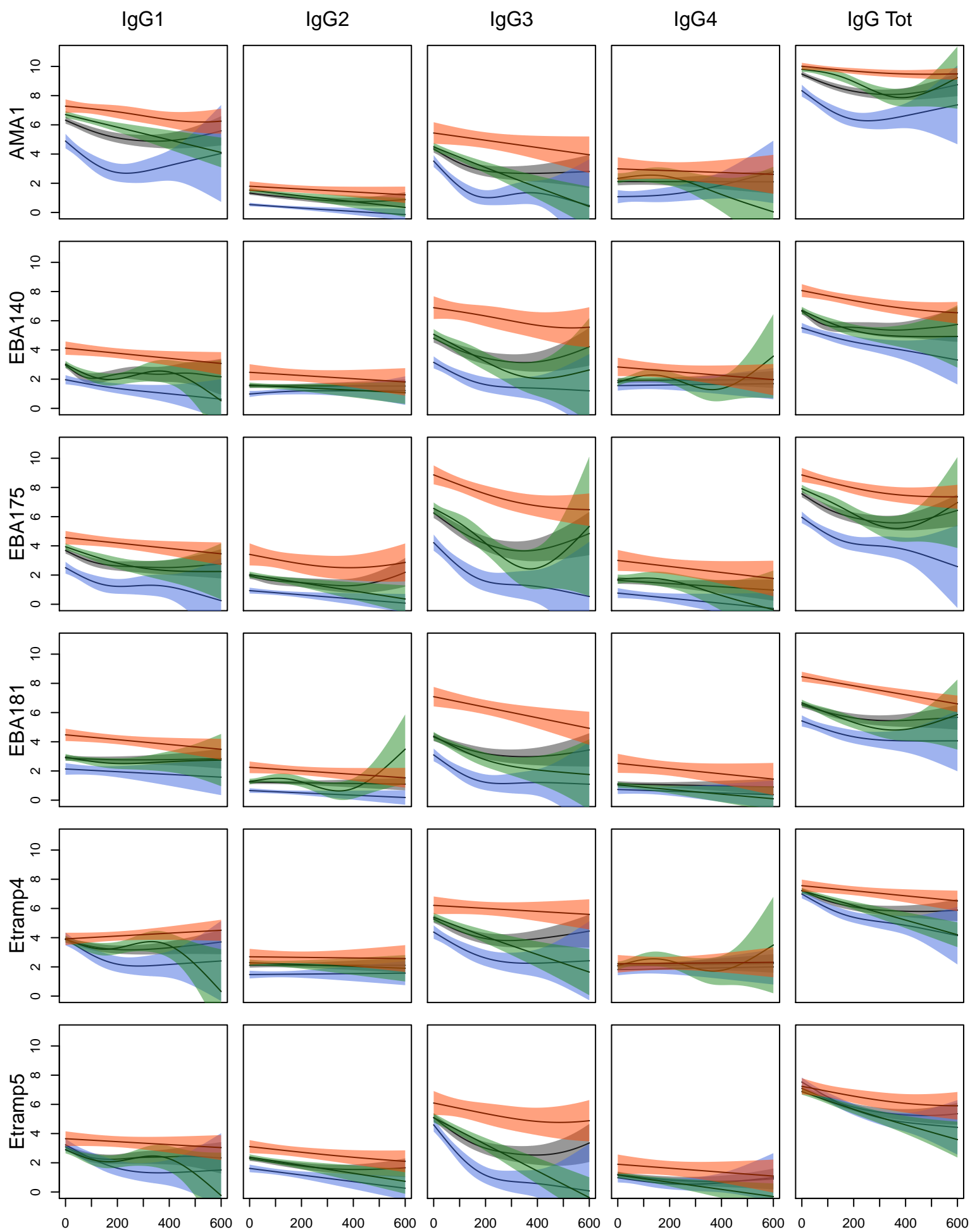

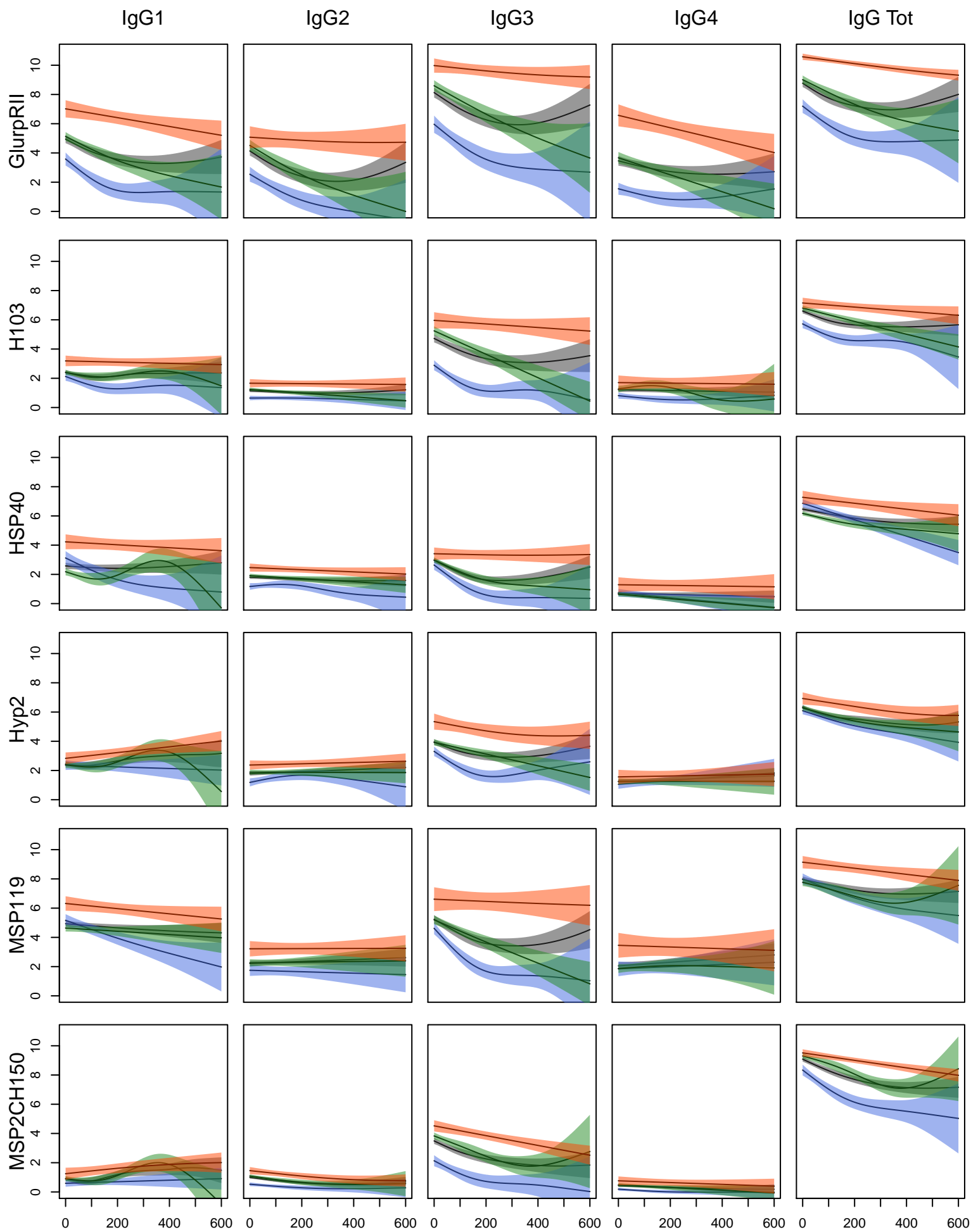

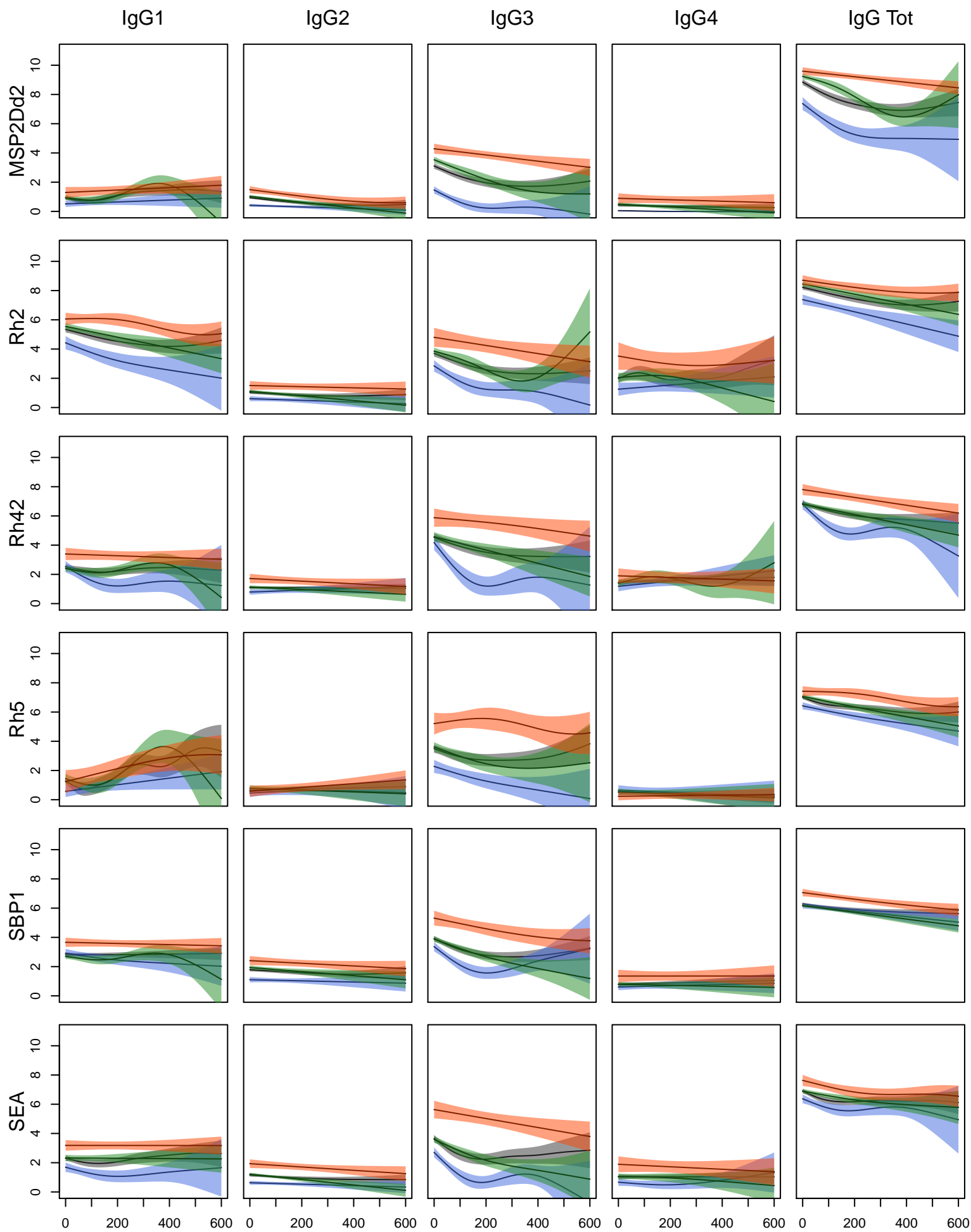

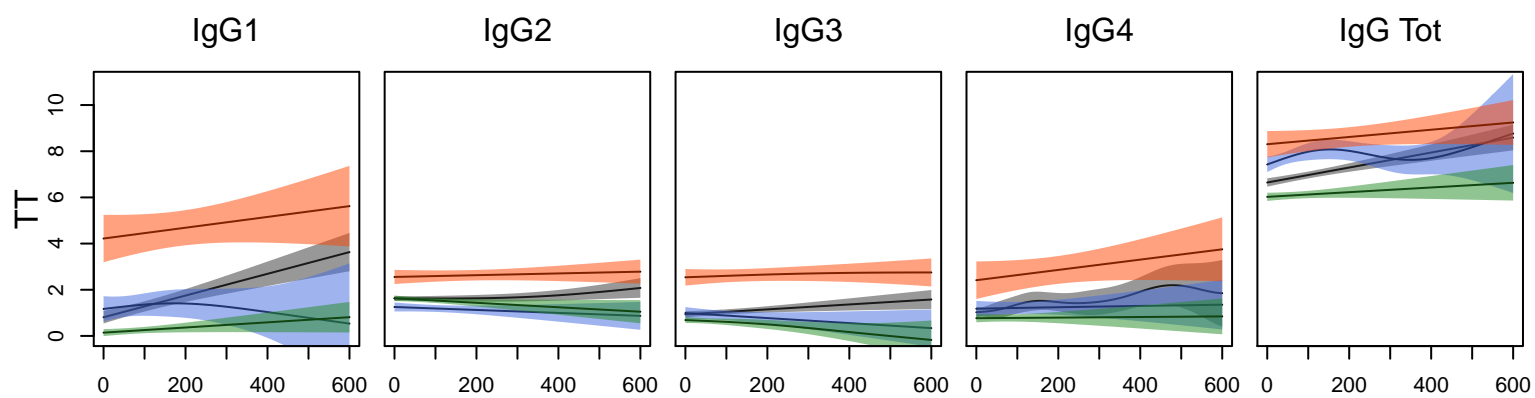

Supplement: Supplementary Figure 2 — Changes in total IgG and subclasses 1 – 4 with age. Total IgG and IgG1 – 4 for 19 P falciparum antigens and TT was measure in a multiplex bead array assay. Plots of mean Log10 MFI against days since last Infection, modeled using generalized additive models (GAMS), shaded areas represent 95% confidence interval. [file Image_2.pdf]

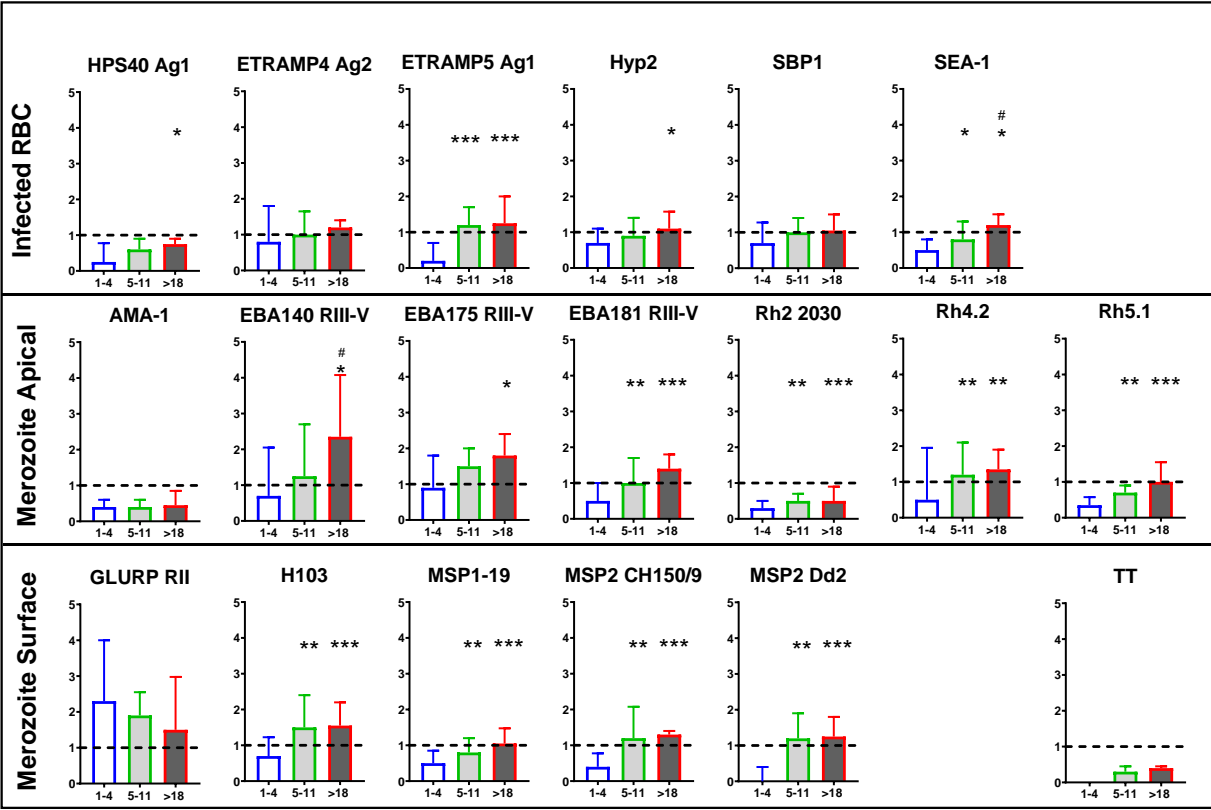

Supplement: Supplementary Figure 3 — Changes in total IgG3/IgG1 ratio with age. Total IgG and IgG1 – 4 for 19 P falciparum antigens and TT was measure in a multiplex bead array assay. Blue =1-4 years (N= 40), Green = 5-11 (N= 92) and Red = >18 years (N= 28). Gray = average (N=160) Ns = not significant, * = p= 0.05 – 0.01, ** p = 0.009 – 0.0001 *** p>0.0000. [file Image_3.pdf]
