# Supplementary material for: Impact of a Rapid Decline in Malaria Transmission on Antimalarial IgG Subclasses and Avidity
Source: Front Immunol. 2021 Jan 27;11:576663. doi: 10.3389/fimmu.2020.576663 (PMC7873448; doi:10.3389/fimmu.2020.576663)
Supplement: Supplementary Table 1 — Summary of the P.falciparum Blood stage antigens. [file Table_1.pdf]

Gene ID	Description	Antigen name	Allele	AA	Location	Tag
PF3D7_0501100.1	Heat Shock Protein 40, type II, Antigen1(*KT)	HSP40Ag1	3D7	71-153	iRBC/Gam	GST
PF3D7_0423700	Early Transcribed Membrane Protein 4, Antigen2(*KT)	ETRAMP4Ag2 Ag1Ag2	3D7	76-137	iRBC/PVM	GST
PF3D7_0532100	early transcribed membrane protein 5 Antigen1(*KT)	ETRAMP5Ag1 Ag2Ag1	3D7	26-111	iRBC/PVM	GST
PF3D7_1002000	Plasmodium exported protein (hyp2), unknown function(*KT)	Hyp2	3D7	101-418	iRBC/PVM	GST
PF3D7_0501300	Skeleton-Binding Protein 1(*KT)	SBP1	3D7	1-239	Scht/MC	GST
PF3D7_1021800	Schizont Egress Antigen 1(*KT)	SEA	3D7	810-1083	Scht/MC	GST
PF3D7_1133400	Apical Membrane Antigen 1(52)	AMA-1	FVO	97-546	SpZ/Mer	His_{x6}
PF3D7_1301600	Erythrocyte Binding Antigen-140 Region III-V (53)	EBA140 RIII-V	3D7	770-1064	Mer -M	GST
PF3D7_0731500	Erythrocyte Binding Antigen-175 Region III-V	EBA175 RIII-V	3D7	761-1298	Mer-M	GST
PF3D7_0102500	Erythrocyte Binding Antigen-181 Region III-V (53)	EBA181 RIII-V	3D7	769-1365	Mer -M	GST
PF3D7_1335400	Reticulocyte Binding Protein Homologue 2 (54)	Rh2	D10	2030-2528	Mer-Rh	GST
PF3D7_0424200	Reticulocyte Binding Protein Homologue 4 (55)	Rh4.2	3D7	28-766	Mer-Rh	His_{x6}
PF3D7_0424100	reticulocyte binding protein	Rh5	3D7	26-526	Mer-Rh	C-
PF3D7_1035300	Glutamate Rich Protein R2 (57)	GLURP RII	F32	816-1091	Mer-S	n/a
PF3D7_1036000	Merozoite Surface Protein 11/H101 (58)	H103	3D7	40-243	Mer-S	GST
PF3D7_0930300	19kDa fragment of MSP1 molecule (59)	MSP1_19	Wellcome	1631-1726	Mer-S	GST
PF3D7_0206800	Merozoite surface protein 2, Dd2 allele (60)	MSP2 Dd2	Dd2	22-247	Mer-S	GST
PF3D7_0206800	Merozoite surface protein 2, CH150/9 allele (60)	MSP2 CH150/9	CH150/9	34-215	Mer-S	GST
n/a	Tetanus Toxoid (Non-adsorbed)	TT	n/a	n/a	n/a	n/a
